# Age- and sex-specific smoking trends by birth cohort in Norway, 1978–2024: an analysis of cross-sectional survey data

**DOI:** 10.1016/j.lanepe.2026.101886

**Published:** 2026-10-15

**Authors:** Jacopo Di Silvestre, M. Victoria Salgado, Jihyoun Jeon, Yoonseo Mok, Theodore R. Holford, Tord Finne Vedøy, Kine Pedersen, Emily A. Burger, Rafael Meza

**Affiliations:** a Department of Health Management and Health Economics, University of Oslo, Oslo, Norway; b Massachusetts General Hospital, Center for Health Technology Assessment, Harvard Medical School, Boston, MA, USA; c Centro de Estudios de Estado y Sociedad, Sánchez de Bustamante 27, Ciudad de Buenos Aires, Argentina; d Unidad de Conocimiento Traslacional Hospitalaria Patagónica, Hospital SAMIC El Calafate, Av. Jorge Newbery 453, El Calafate, Santa Cruz, Argentina; e Department of Epidemiology, University of Michigan, 1415 Washington Heights, Ann Arbor, MI, USA; f Department of Biostatistics, Yale School of Public Health, 60 College Street, New Haven, CT, USA; g Department Alcohol, Tobacco, and Drugs, Norwegian Institute of Public Health, Oslo, Norway; h Harvard T.H. Chan School of Public Health, Center for Health Decision Science, Boston, MA, USA; i School of Population and Public Health, The University of British Columbia, 206 East Mall, Vancouver, British Columbia, Canada; j Department of Medicine, Rutgers University, New Brunswick, NJ, USA

**Keywords:** Modeling, Smoking trends, Norway, Tobacco research

## Abstract

**Background:**

Smoking is the primary cause of lung cancer, the leading cause of cancer-related mortality in Norway. To inform disease modeling and tobacco control policies, including lung cancer screening, we estimated sex-specific, birth-cohort smoking behaviors among Norwegians born between 1950 and 2000 using national survey data.

**Methods:**

We analyzed nationally representative cross-sectional survey data from 1978 to 2024 (n = 78,282) to reconstruct smoking trends and smoking intensity in Norway. We used a previously established Age–Period–Cohort modeling framework to estimate cohort-specific prevalence of daily ever-smoking, probabilities of smoking initiation and cessation, prevalence of daily current-smoking, and average cigarettes smoked per day (CPD). Analyses were conducted separately by sex assigned at birth for cohorts born between 1950 and 2000.

**Findings:**

Daily ever-smoking prevalence peaked in the 1950 birth cohort, reaching 70% (90% CI 66%–91%) among males and 66% (90% CI 55%–89%) among females, alongside similar peaks in smoking initiation probabilities, 13% (90% CI 12%–24%) for males and 12% (90% CI 10%–23%) for females. Smoking cessation probabilities increased with age within each birth cohort, rising from approximately 0% to 5% over 40 years in earlier cohorts, compared with an increase from about 5% to 15% over 20 years from the 1965 birth cohort onward. Smoking intensity peaked at 15 CPD among males and 12 CPD among females. Although smoking intensity declined in both sexes after the 1975 birth cohort, it remained higher among males, with peak model-estimated CPD decreasing from approximately 13.5 to 10 cigarettes in males and from 11.5 to 7.5 cigarettes in females between the 1980 and 2000 birth cohorts.

**Interpretation:**

Smoking behavior in Norway has changed across generations, with declining smoking initiation rates, increased smoking cessation, and lower cigarettes per day. Understanding these trends provides essential inputs to inform tobacco control and lung cancer prevention policies in Norway.

**Funding:**

Norwegian Cancer Society, National Cancer Institute of the National Institutes of Health, U.S. Food and Drug Administration.

**Research in context**

**Evidence before this study**

Smoking is the primary cause of lung cancer, which remains the leading cause of cancer-related death globally and in Norway. Specifically in Norway, lung cancer causes approximately 2200 deaths annually, accounting for 19.3% of all cancer deaths in 2022 and ranking as the fifth leading cause of death overall. Most surveillance systems report smoking prevalence, but prevalence alone does not capture the underlying dynamics of smoking initiation, cessation, and intensity (defined as cigarettes per day, CPD). These measures are essential inputs for disease simulation models used to project future disease burden and evaluate tobacco control policies. We searched PubMed and Google Scholar on September 6, 2026, with no date restrictions, using terms related to Norway, smoking, initiation, cessation, intensity, age, and birth cohort. We also reviewed relevant reports and statistics from Statistics Norway (SSB) and the Norwegian Institute of Public Health (FHI), and references of identified studies. Although previous studies described smoking prevalence and trends in Norway, we identified no age- and birth-cohort-specific estimates of smoking initiation, cessation, and intensity.

**Added value of this study**

Using nationally representative cross-sectional survey data we estimated age- and birth-cohort-specific smoking behaviors among Norwegians born between 1950 and 2000, including daily smoking prevalence, initiation and cessation probabilities, and smoking intensity measured as cigarettes per day. We found substantial cohort differences, with declining smoking initiation and prevalence and increasing daily smoking cessation probabilities in more recent birth cohorts. To our knowledge, this is the first study to develop such a comprehensive analysis of smoking behavior by birth cohort and to estimate age- and cohort-specific smoking initiation and cessation probabilities in Norway.

**Implications of all the available evidence**

Smoking prevalence alone provides an incomplete picture of population smoking behavior. By quantifying smoking initiation, cessation, and intensity (cigarette consumption) across birth cohorts, our study provides a more comprehensive understanding of smoking dynamics across generations. Our findings provide novel population-based measures for Norway that can serve as key inputs for health economic evaluations and disease simulation models. These estimates can support assessment of the long-term health impacts of tobacco control and cancer prevention strategies, including screening and smoking cessation interventions.

## Introduction

Smoking is the primary cause of lung cancer, which remains the leading cause of cancer-related death globally and in Norway.^1^ Specifically in Norway, lung cancer causes approximately 2200 deaths annually, accounting for 19.3% of all cancer deaths and ranking as the fifth leading cause of death overall.^2^^,^^3^ The economic burden is also significant, with lung cancer treatment costs exceeding €3 billion annually across Europe.^4^ Smoking also substantially increases the risk of other cancers, chronic respiratory diseases such as chronic obstructive pulmonary disease (COPD), and cardiovascular diseases imposing additional substantial health and economic costs on society.^5^^,^^6^ Consequently, tobacco control policies in Norway are designed to reduce the overall health and economic burden of smoking, not only lung cancer.

Norway has a history of smoking-reduction policies (see Supplementary Fig. S1); most notably, the 1975 Tobacco Act, the 1988 Clean Air Act, and the 2004 nationwide ban on indoor smoking. More recently, in 2017, Norway introduced standardized packaging for all tobacco products and extended the smoke-free legislation to include e-cigarettes.^7^ While the prevalence of daily smokers is at an all-time low among younger generations in Norway, it was considerably higher among people from earlier cohorts, such as the 1950 and 1960 birth cohorts,^8^ which are now reaching their peak ages of lung cancer and other tobacco-related diseases. Consequently, the Norwegian government is currently evaluating a national lung cancer screening program coupled with smoking cessation interventions. As over 90% of lung cancers occur in current or former smokers,^9^ understanding generational smoking patterns is essential for effective tobacco control, capacity planning, and evaluating the potential impact of a national lung cancer screening program. That is, evaluations need to include a better understanding of both past and current smoking behaviors, and how smoking patterns have changed over time and by birth cohort. Importantly, deconstructing cross-sectional smoking into the underlying behaviors of smoking initiation, cessation, and intensity of smoking in terms of cigarettes per day (CPD) is essential for making accurate projections about the long-term impact of tobacco use and evaluating the effects of potential public health interventions, both within and beyond lung cancer.^10^^,^^11^

Public agencies such as Statistics Norway have collected information on smoking behavior among the population since 1973, enabling the reconstruction of smoking trends across generations.^12^, ^13^, ^14^ Age-Period-Cohort (APC) models applied to the cross-sectional panel data have been used to reconstruct smoking histories despite biases in retrospective surveys,^15^, ^16^, ^17^, ^18^, ^19^, ^20^, ^21^ including Norway.^22^^,^^23^

Although trends in current and ever daily smoking prevalence by calendar year and by birth cohort in Norway have been well characterized,^22^ less is known about the smoking prevalence of more recent birth cohorts, or the rates of smoking initiation, cessation, and intensity (i.e., CPD), which vary by birth cohort. While a previous Norwegian report^24^ has analyzed smoking behavior in Norway, we extended this work by estimating age-specific smoking initiation and cessation probabilities and smoking intensity (CPD) for each birth cohort.

Quantification of smoking patterns by birth cohort, specifically age- and cohort-specific probabilities of smoking initiation and cessation and smoking intensity (CPD), can be used as inputs in disease simulation modeling, rather than for direct policy evaluation, to quantify the health and economic consequences of smoking and its control efforts. Examples of these analyses have been undertaken by the U.S. Cancer Intervention and Surveillance Modeling Network (CISNET) Lung Working Group (LWG). These models evaluated the impact of tobacco control policies, low-dose CT screening, and treatment interventions on lung cancer incidence and mortality.^12^^,^^13^^,^^25^, ^26^, ^27^ Their work has been instrumental in shaping lung cancer control efforts in the U.S., including informing the 2021 U.S. Preventive Services Task Force screening guidelines.^26^

To support public health planning and the evaluation of future tobacco control and lung cancer screening strategies in Norway through cost-effectiveness analyses, we aimed to quantify age-, sex- (hereafter, “sex” refers to sex assigned at birth) and birth-cohort-specific smoking histories for Norwegians born between 1950 and 2000. Using national cross-sectional surveys, we estimated the prevalence of current and ever daily smoking, the probabilities of initiation and cessation, and the distribution of CPD for each birth cohort and sex among daily current smokers.

## Methods

### Smoking behavior data

We obtained data from Statistics Norway (SSB) Travel and Holiday Surveys (Omnibus)^28^ and Smoking Habits Survey (Drug Survey in 2023–2024)^29^ with both surveys providing data annually for each year from 1973 to 2024 (see Supplementary Table S1 for more details). As the data were anonymous, ethical approval from the Regional Committees for Medical and Health Research Ethics (REK) and informed consent were not required. For each respondent and survey year, we used anonymous self-reported information on cigarette smoking to classify individuals as current, former, or never daily smokers (classification algorithm in the Supplementary Material). We then calculated cross-sectional estimates of smoking prevalence by age, sex, and calendar year. The surveys also included questions on tobacco use, such as age at smoking initiation and cessation, and usual number of cigarettes smoked per day (CPD). We used these items to retrospectively reconstruct individual smoking histories (ages at initiation and cessation and smoking intensity) and to derive age-specific initiation, cessation, and CPD patterns by birth cohort and sex. All cross-sectional surveys are public and nationally representative,^30^ with similar demographic characteristics (see Supplementary Table S3 for distributions of sex and ages in each year) and were conducted from samples of people aged 16–79 years living in Norway (aged 16–74 years prior to 1992) at each survey year^29^ (see Supplementary Table S4 for a complete overview of the number of respondents every year).

The Norwegian Institute of Public Health (NIPH) created an initial dataset using the survey data (owned by SSB). The dataset included variables on age, year of survey, birth year (calculated as year of survey–age), sex, and smoking history. We used smoking initiation and cessation ages to calculate year at cessation, years since cessation, and year of initiation. We grouped CPD into categories (1–5, 6–10, 11–15, 16–20, 21–25, 26–30, 31–35, 36–40, 41–45, and 46+) and included survey weights for population adjustment.

Using the initial dataset, which included 226,698 observations, we subset our sample to include the fourth quarter data only (removed 115,765 observations, 51.1%), because information on smoking onset and cessation, and CPD were only available for this quarter. We removed missing values (0.3%) from tobacco use questions and removed surveys administered prior to 1978 (25,164 observations, 11.1%), as they did not include information on the year of smoking cessation variable. We excluded an additional 7413 observations (3.3%) that reported occasional smoking (analysis restricted to daily smokers); finally, we removed some inconsistencies, e.g., observations where the respondents’ age at start smoking was recorded after the age at cessation, or any missing values in these two variables (removed 74 observations, 0.03%). The dataset was complete for all variables included in the analyses, and no additional methods for handling missing data were required. We used this final sample (n = 78,282) to estimate age-specific smoking prevalence, probabilities of initiation and cessation, and intensity by age, sex, and birth cohort among daily smokers. Here, cessation refers specifically to cessation of daily smoking, which encompasses complete abstinence from smoking and transition to non-daily smoking.

For all analyses, we used cross-sectional survey data available for the period 1978–2024 and for ages 16–79 years (16–74 years before 1992), as summarized in Supplementary Fig. S2. For example, the 1950 birth cohort was represented in the 2000 survey by individuals aged 50 years. As survey participation requires a minimum age of 16 years, the most recent cohort represented was born in 2008, and therefore had only a very limited observation period.

### Statistical analysis

#### Overview of modeling approach

We followed a previously established APC methodology by the CISNET LWG,^14^^,^^27^ consisting of a compartmental model for smoking history, which has been applied using survey data from the United States, Canada, Brazil, and Argentina.^14^^,^^27^^,^^31^^,^^32^ The analysis was conducted in R version 4.5.2.^33^

#### Smoking states and transitions

We modeled three mutually exclusive daily smoking states for daily cigarette smoking at each age: never smoker, current smoker, and former smoker (Supplementary Fig. S3). Individuals can transition from never smoker to current smoker (initiation) and from current smoker to former smoker (cessation); after cessation, they remain former smokers. No transitions from former back to current are allowed in this model. We estimated annual transition probabilities conditionally on a hypothetical scenario assuming no transitions to death,^27^ isolating smoking behavior dynamics from mortality-related effects.

#### Implementation of the APC model

We applied APC models to estimate the probabilities of age-specific smoking parameters by birth cohort (1950–2000) and sex in Norway. Age effects capture the pattern of smoking over the life-course, period effects capture changes common to all cohorts (e.g., policy changes), and cohort effects capture differences between birth cohorts. Outcomes included initiation and cessation probabilities, prevalence of ever, current, and former smoking, as well as smoking intensity (CPD) among daily current smokers.^27^ The estimates from these previous analyses were well validated against the observed data (see Supplementary Material), and therefore, the results can be interpreted with high certainty.^34^

We estimated the initiation probabilities as the conditional probability that an individual in a cohort started smoking at a certain age, at least 8 years (i.e., smoking initiation probability of zero before 8 years old), given the non-smoking status the year before.^27^ We used these individuals to estimate the cumulative proportion of daily ever smokers by age using a life table approach, recognizing potential bias due to differential mortality by smoking status.^35^ Starting at age 15 years, we parameterized transitions from current to former smokers using cessation probabilities, conditional on being alive and currently smoking.^27^ Because of survey limitations, individuals were classified as former smokers only if at least two years had elapsed since the reported age of cessation, without modelling later relapse or multiple transitions between smoking states; sensitivity analyses using alternative thresholds were performed (see Supplementary Material). These ages were chosen to match prior APC applications and because initiation before age 8 and sustained daily smoking before age 15 are extremely rare in population data. For each cohort, we calculated the cumulative proportion of daily current smokers who had not ceased by a certain age, calculated as one minus the product of the conditional probabilities of not ceasing. We assumed cessation probabilities were independent of age at initiation, smoking intensity, and other factors such as socioeconomic status. As most smoking initiation occurs in a narrow age range, the variation in time since initiation has a limited influence on mortality trends as cohorts age.

We defined daily smoking age-specific initiation probabilities among individuals who had never smoked and age-specific cessation probabilities among daily current smokers. We scaled initiation probabilities by cohort to match estimates of ever smoking prevalence at age 30 years^27^ (Supplementary Material).

#### Sensitivity analysis

We quantified sample uncertainty around the APC-derived smoking history estimates, through a non-parametric bootstrap following a previously described approach.^34^ We generated 1000 bootstrap datasets by resampling individuals from the original analytic dataset with replacement, refitted the full APC model to each bootstrap sample, and derived the corresponding estimates of smoking initiation, cessation, and smoking prevalence. Empirical 90% uncertainty intervals were then determined finding the 5th and 95th percentiles of the bootstrap distribution for each age-, birth cohort-, and sex-specific estimate.

#### Role of the funding source

The funders had no role in the study design, in the collection, analysis, and interpretation of data; in the writing of the report; and in the decision to submit the article for publication.

## Results

### Descriptives

Among the 78,282 respondents included in our sample, the proportion of respondents reporting never (daily) smoking increased steadily from the earliest to the most recent birth cohorts, with higher proportions of reported daily never smokers observed among more recent cohorts within every age group (Supplementary Table S2). For example, among females, the proportion of daily never smokers increased from 47.5% in individuals aged 15–19 years in the 1960 birth cohort to 100% in the 2000 birth cohort, among the same age group. Similarly, for males, the proportion increased from 42.3% in the 1960 birth cohort to 97.4% in the 2000 birth cohort.

### Smoking trends by birth cohort and sex

We identified distinct temporal patterns in smoking behaviors of daily smokers across birth cohorts by sex. For both sexes, daily ever-smoking prevalence declined across successive birth cohorts with the highest levels observed in the 1950 cohort, where the prevalence reached 66% (90% CI 55%–89%) among females (Fig. 1—left panel) and 70% (90% CI 66%–91%) among males (Fig. 1—right panel). In the bootstrap analysis, uncertainty intervals were generally wider for earlier birth cohorts and older ages, while narrower intervals were observed for more recent birth cohorts (Supplementary Figs. S9–S12). Despite these similar overall declines, important sex-specific differences emerged across cohorts with females exhibiting a catch-up pattern in ever-smoking prevalence. Although female prevalence was lower than that of males in the 1950 and 1955 cohorts, it exceeded male prevalence in the 1960, 1965, 1970, and 1975 cohorts. In the 1980 cohort, prevalence levels converged between sexes, after which ever-smoking prevalence among males remained higher than that of females in all subsequent birth cohorts.

**Fig. 1:**
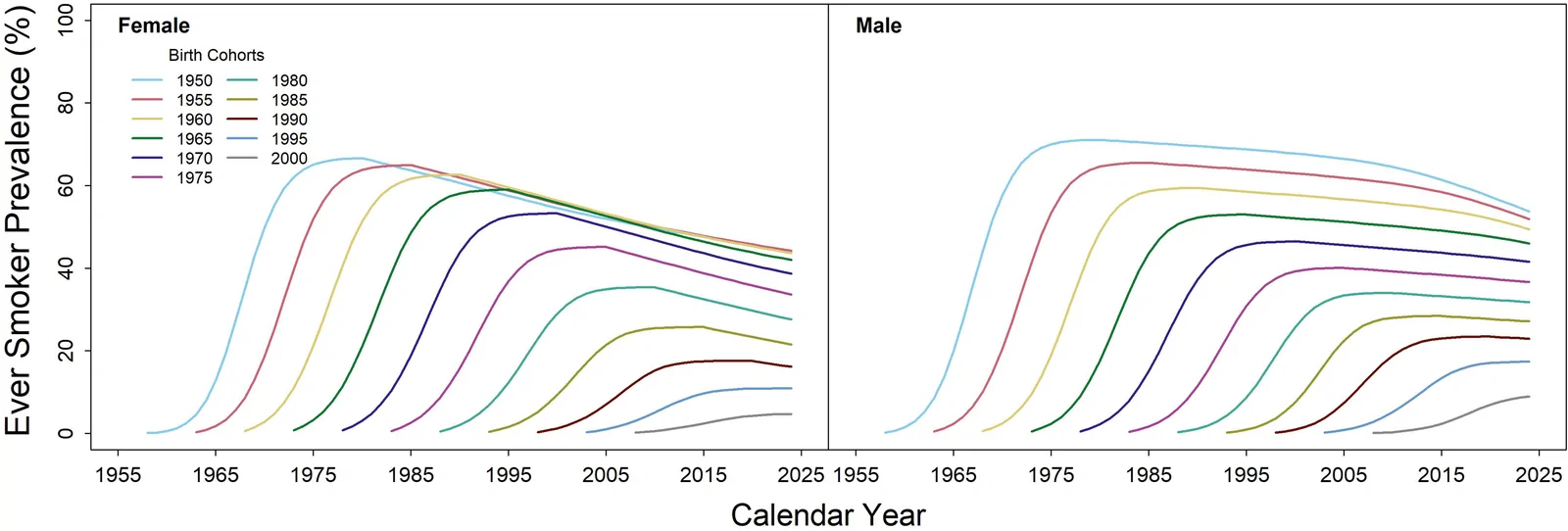
Age-specific daily ever-smoking prevalence for selected birth cohorts, defined by year of birth, at 5-year intervals from 1950 to 2000, by sex. Each colored line represents a specific birth cohort, beginning with individuals born in 1950 (light blue line). The progression of each line along the horizontal axis illustrates how the prevalence of daily ever smoking changes as the corresponding birth cohort ages. We assumed the probability of smoking initiation to be zero before age 8 years; therefore, daily ever-smoking prevalence begins to increase from age 8 years.

For both sexes, smoking initiation probabilities followed a bell-shaped pattern, increased with age, peaked around age 19 years across birth cohorts, and then decreased steeply. The highest initiation probabilities occurred in the 1950 birth cohort, at 12% (90% CI 10%–23%) among females (Fig. 2—left panel) and at 13% (90% CI 12%–24%) among males (Fig. 2—right panel); thereafter, initiation probabilities declined steadily, with higher values observed among females in the 1960–1975 birth cohorts, while initiation probabilities among males remained higher in cohorts born after 1980.

**Fig. 2:**
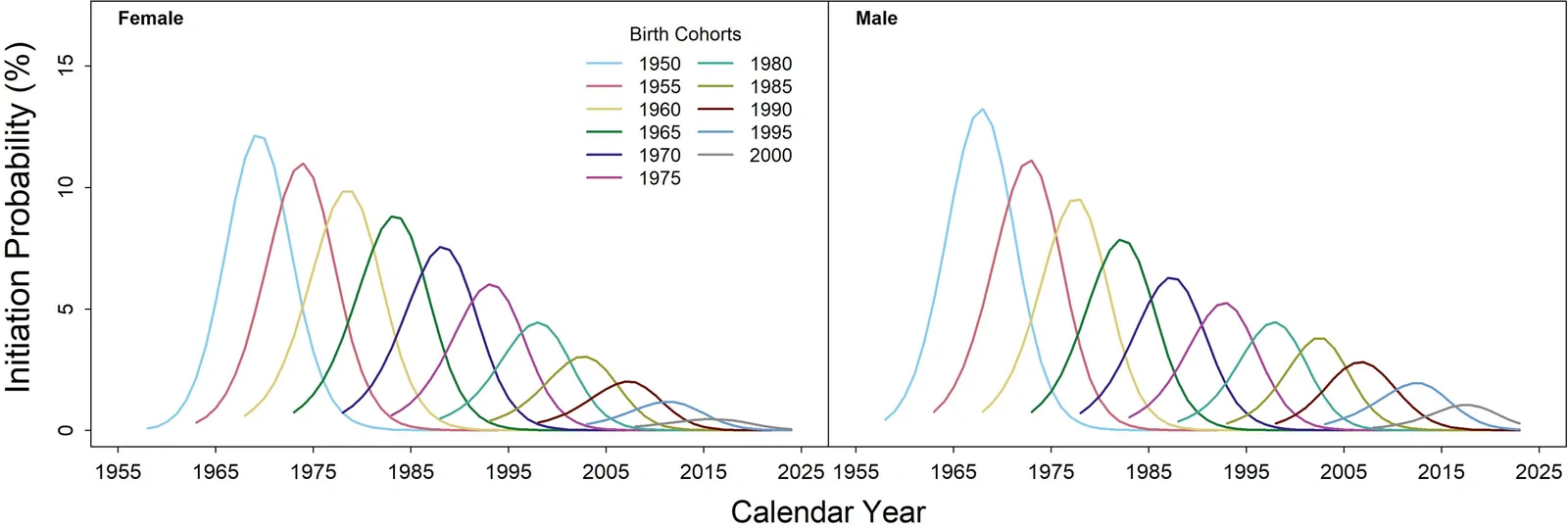
Age-specific daily smoking-initiation probabilities (percentage) for selected birth cohorts, defined by year of birth, at 5-year intervals from 1950 to 2000, by sex. Each colored line represents a specific birth cohort, beginning with individuals born in 1950 (light blue line). The progression of each line along the horizontal axis illustrates how the probability of smoking initiation changes as the corresponding birth cohort ages. We assumed initiation probabilities to be zero before age 8 years.

Smoking cessation probabilities increased with age within each birth cohort, with earlier cohorts showing only small increases in cessation by age (probabilities increasing from 0% to 5% over 40 years). However, beginning with the 1965 cohort, both sexes demonstrated sharper increases in smoking cessation (probabilities increasing from 5% to 15% over 20 years) (Fig. 3). Males initially exhibited higher cessation probabilities than females in earlier cohorts; however, females surpassed males in the more recent birth cohorts.

**Fig. 3:**
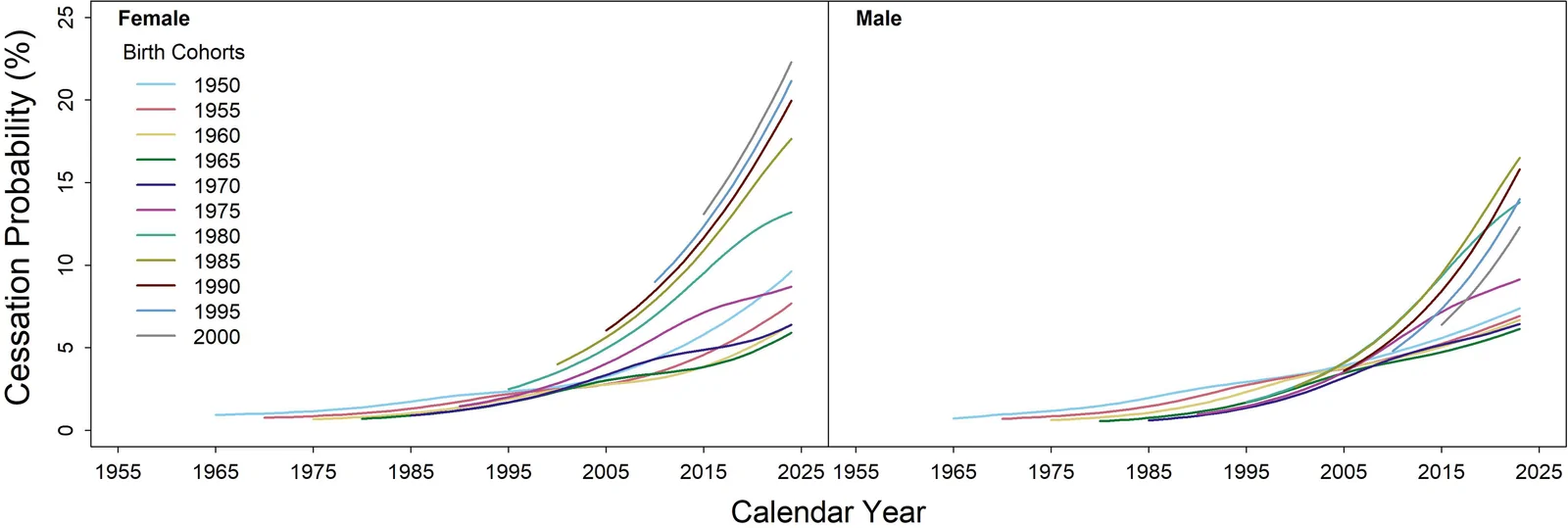
Age-specific daily smoking-cessation probabilities (percentage) for selected birth cohorts, defined by year of birth, at 5-year intervals from 1950 to 2000, by sex. Each colored line represents a specific birth cohort, beginning with individuals born in 1950 (light blue line). The progression of each line along the horizontal axis illustrates how the probability of smoking cessation changes as the corresponding birth cohort ages. We assumed cessation probabilities to be zero before age 15 years.

Current smoking prevalence followed a pattern similar to ever-smoking prevalence, but declined more rapidly, and was less sustained over time. The prevalence peaked in the 1950 birth cohort at 63% (90% CI 61%–79%) among males and 58% (90% CI 51%–75%) among females (Fig. 4), with modeled estimates closely matching the empirical data (Supplementary Figs. S4 and S5).

**Fig. 4:**
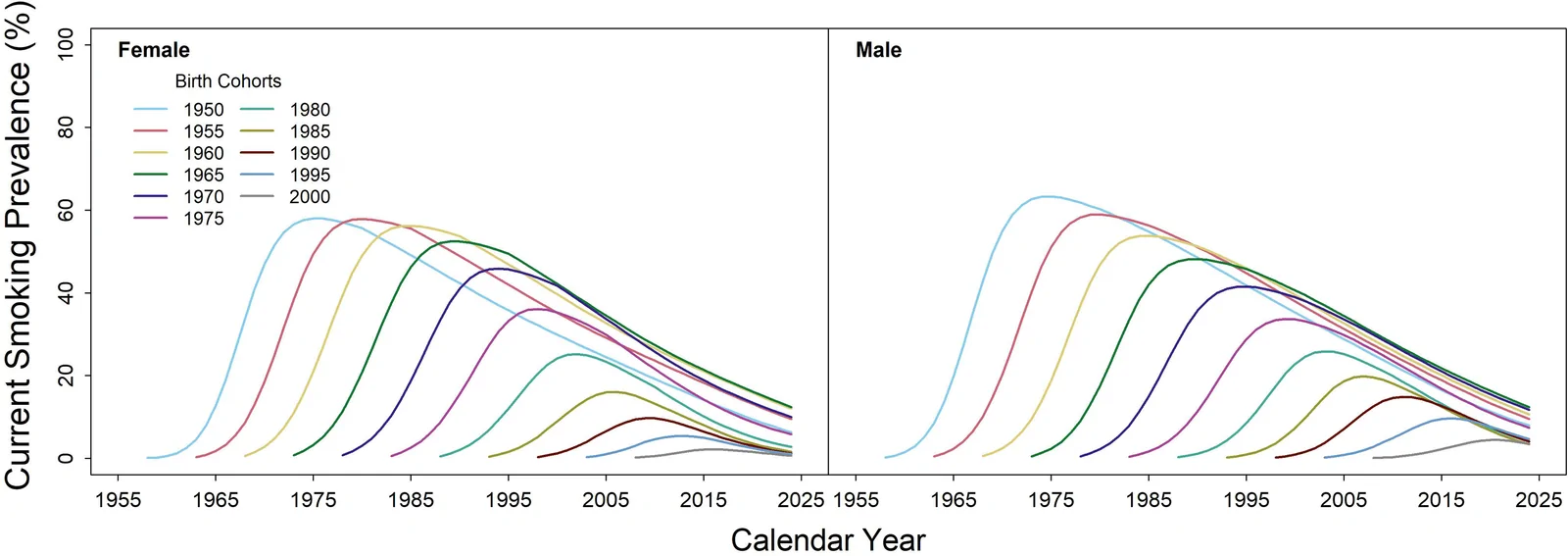
Age-specific daily current-smoking prevalence for selected birth cohorts, defined by year of birth, at 5-year intervals from 1950 to 2000, by sex. Each colored line represents a specific birth cohort, beginning with individuals born in 1950 (light blue line). The progression of each line along the horizontal axis illustrates how the prevalence of daily current smoking changes as the corresponding birth cohort ages. We assumed initiation probabilities to be zero before age 8 years; therefore, daily current-smoking prevalence begins to increase from age 8 years.

Finally, when we estimated smoking intensity measured by age-specific CPD in each cohort among daily smokers, we found relatively stable trends across early cohorts peaking at 12 CPD for females and 15 CPD for males (Supplementary Fig. S6). Age effects produced a bell-shaped curve in both groups, though wider in females, reflecting more gradual increases and declines in smoking intensity by age, and narrower in males, indicating faster changes in smoking intensity. Smoking intensity was higher among males than females, with peak model-estimated CPD declining from approximately 13.5 to 10 cigarettes among males and from 11.5 to 7.5 cigarettes among females between the 1980 and 2000 birth cohorts. Modeled smoking intensity corresponded with the empirical data (Supplementary Figs. S7 and S8).

Bootstrap-derived uncertainty intervals for smoking initiation, cessation, and ever/current smoking prevalence are presented in Supplementary Figs. S9–S12 and Supplementary Tables S5 and S6.

## Discussion

We used nationally representative cross-sectional smoking behavior data collected over 45 years to provide the first estimates of smoking initiation and cessation probabilities, as well as CPD, by birth cohort in Norway. These measures capture the balance between initiation, cessation, and smoking intensity, offering a more comprehensive understanding of smoking dynamics across generations. These measures are essential inputs for our planned disease simulation modeling, which will be used to inform tobacco control planning and to evaluate the cost-effectiveness of lung cancer prevention strategies, including screening.

We found that daily smoking behaviors varied across birth cohorts and by sex, with daily smoking prevalence being generally higher in males compared to females, and with sustained declines in both groups. These sex-specific patterns are consistent with the tobacco epidemic model, in which smoking uptake and prevalence historically increased earlier among men than among women, followed by a later rise among women and subsequent convergence between the sexes as the epidemic progressed.^36^ Similar cohort- and sex-specific patterns were evident for daily smoking initiation and cessation, with females exhibiting higher cessation probabilities than males in more recent birth cohorts. Daily smoking intensity, measured by CPD, remained consistently higher among males compared with females despite declines in more recent cohorts.

Our findings are broadly consistent with previous studies estimating sex-specific differences in smoking patterns in Norway, including an overall higher prevalence for males, a catch-up effect of females and subsequent convergence in more recent cohorts,^22^^,^^23^ despite using a different analytic approach. The catch-up effect and overall prevalence rates shown in our analysis confirm trends in the ever-smoking prevalence observed in earlier studies, but our results add to existing knowledge by quantifying initiation, cessation, and intensity across birth cohorts. Initiation followed a bell-shaped distribution, peaking in the 1950s for both sexes before declining steadily. Cessation increased only modestly in early cohorts but rose sharply from the mid-1960s, with females surpassing males in more recent cohorts. CPD was stable across cohorts and declined after birth cohort 1975, with consistently higher CPD values among males. These findings align with international evidence,^14^^,^^16^^,^^27^^,^^32^ while introducing new population estimates for Norway.

The observed declines in current and ever smoking prevalence and smoking intensity are likely attributable to both behavioral shifts and Norway’s tobacco control policies.^37^ It’s worth noting that the sharp decline in the youngest cohorts may be influenced by the substitution of snus for cigarettes in Norway. Snus use has increased markedly in Norway since around 2000 and may have contributed to declining smoking initiation and higher cessation, particularly in cohorts born after 1980, although its causal impact appears uncertain^38^ (Supplementary Figs. S13–S14, and Supplementary Section VI).

This study has some limitations, which include the modest sample sizes, the cessation information restricted to surveys conducted after 1978, and the absence of data on initiation and cessation for occasional smokers. Excluding occasional smokers may result in underestimation of smoking initiation probabilities and overall smoking prevalence as occasional smokers who have initiated smoking are classified as never daily smokers. Similarly, our cessation probabilities should be interpreted as probabilities of cessation of daily smoking, which encompasses both complete smoking cessation and transition to non-daily smoking. As our data did not allow us to distinguish individuals who transitioned from daily to occasional smoking from those who stopped smoking entirely, both transitions were grouped together. Consequently, the estimated cessation probabilities may overestimate the probability of complete smoking cessation. Although the proportion of individuals reporting occasional smoking was relatively small (8.6%), occasional smoking has been associated with increased mortality in Norwegian cohort studies,^39^ suggesting that its exclusion may still lead to underestimation of the population burden attributable to smoking. E-cigarette use was also limited and occurred mostly in recent years.^40^ While this APC modeling approach captures initiation, cessation, and smoking intensity, it does not allow causal attribution of the observed patterns to specific policies or events. Additionally, cessation probabilities were not influenced by age at initiation, prior cessation history, or smoking intensity. Our model did not allow relapse from former to current daily smoking, potentially overestimating sustained cessation, especially in the younger age groups where relapse is more frequent. More comprehensive models could incorporate stochastic processes, include transitions between additional smoking states (such as former smokers), account for covariates that affect transition rates (for example, smoking intensity and socioeconomic status), and use mortality rates specific to smoking status. Because our methodological framework did not incorporate age at initiation, prior cessation history, smoking intensity, or socioeconomic status into the cessation probabilities, we could not assess how these factors might influence cessation. Future work should explore potential heterogeneities of smoking intensity-dependent cessation probabilities. The mortality adjustment assumes negligible effects before age 30 and a constant multiplicative bias; given the substantial excess mortality among smokers observed in Norwegian cohort studies,^41^ residual survivorship bias may nevertheless remain, particularly at older ages and in earlier cohorts.

Sampling variability in the APC-derived smoking history parameters is a potential limitation. To assess uncertainty, we performed a non-parametric bootstrap analysis with 1000 resampled datasets. Although uncertainty bounds were relatively wide in some birth cohorts and narrower for later birth cohort, they did not materially alter the qualitative age-, cohort-, or sex-specific patterns. Nonetheless, smaller differences between sexes or adjacent cohorts, such as the modest gap in peak ever-smoking prevalence in the 1950 birth cohort, should be interpreted cautiously given the overlapping uncertainty intervals.

Strengths of this study include the use of nationally representative data across multiple decades, the introduction of novel smoking indicators and the methodological approach that has been applied successfully in other countries, supporting the validity of our estimates and ensuring comparability across contexts. Although based on repeated cross-sectional data, this approach is consistent with established APC frameworks, while individual–level transitions cannot be directly observed.^42^^,^^43^

In conclusion, daily smoking behaviors in Norway have undergone substantial cohort- and sex-specific shifts. The daily ever and current smokers’ prevalence exhibited a pronounced catch-up effect among females, with initially lower prevalence than males in the 1950 and 1955 birth cohorts, higher prevalence in the 1960–1975 cohorts, convergence in the 1980 cohort, and a subsequent return to higher prevalence among males in cohorts born after 1985. We observed declines in initiation probabilities and smoking intensity, alongside rising cessation probabilities across successive birth cohorts. These descriptive, cohort-specific estimates of initiation, cessation, and cigarettes per day provide detailed smoking-history inputs for future disease modelling and health-economic analyses in Norway but should be interpreted in light of the study’s limitations, including the reconstruction of histories from repeated cross-sectional data, exclusion of occasional smokers, and potential survivorship bias.

## Contributors

Conceptualization: JDS, EAB, MVS, YM, JJ, TRH, RM, TFV.

Formal analysis: JDS.

Methodology: JDS, MVS, YM, JJ, TRH, RM.

Supervision: EAB, RM.

Writing: JDS.

Review & editing: JDS, KP, MVS, YM, JJ, TRH, RM, TFV.

JDS and TFV had access to and verified the data. Authors’ statement: all authors had full access to all the data in the study and accept responsibility to submit for publication.

## Data sharing statement

The Norwegian data used in this analysis cannot be shared with anyone outside the study researchers by law. The authors were provided access to anonymous survey data from Statistics Norway (SSB) for research purposes. As the data were anonymous, ethical approval from the Regional Committees for Medical and Health Research Ethics (REK) was not required. This was confirmed with the Norwegian Agency for Shared Services in Education and Research (Sikt). However, these data are publicly available for research purposes and can be obtained by submitting a new application directly to the relevant data holders.

## Declaration of interests

All authors declare that they have no conflicts of interest.
