# Supplementary material for: Age- and sex-specific smoking trends by birth cohort in Norway, 1978–2024: an analysis of cross-sectional survey data
Source: Lancet Reg Health Eur. 2026 Oct 15;71:101886. doi: 10.1016/j.lanepe.2026.101886 (PMC13694876; doi:10.1016/j.lanepe.2026.101886)
Supplement: Supplementary file 1 [file mmc1.docx]

**Smoking patterns by birth cohort in Norway – Supplemental material**

Table of Contents

[I. Data sources, sample, policies	2](#_Toc232928403)

[II. Methodology Overview for the Age-Period-Cohort Model	7](#_Toc232928404)

[III. Comparison of current smoking prevalence and mean CPD as reported by surveys vs model predictions and CPD analysis	11](#_Toc232928405)

[IV. Uncertainty Analysis	13](#_Toc232928406)

[V. Association between daily smoking and daily snus use	15](#_Toc232928407)

[VI. Overall snus use and current smoking	16](#_Toc232928408)

[VI. References	17](#_Toc232928409)

# I. Data sources, sample, policies

The Travel and Holiday Surveys[1] (Omnibus) and what is often referred to as the Smoking Habits Survey[2] are parts of several nationally representative surveys carried out by Statistics Norway (SSB). The Travel and Holiday Survey was administered yearly from 1992 to 2024, while the questions commonly associated with the “Smoking Habits Survey” have appeared in different physical survey vehicles over time: in the Labour Force Survey (4th quarter) from 1973 to 1991, in the Travel and Holiday Surveys (4th quarter) from 1992 to 2022, and in 2023 and 2024 as part of the Drug Survey conducted in the 2nd quarter.

The main purpose of the Travel and Holiday Survey was to map Norwegians’ travel habits and to serve as a data source for other official statistics. It also included modules on topics such as smoking, attitudes toward immigration, wood burning, access to ICT, work and family conditions. Some topics were included regularly, while others, such as smoking habits, were included only in specific quarters, but at least once a year.

What is commonly called the “Smoking Habits Survey” began on behalf of the National Tobacco Injury Council. Since 1973, Statistics Norway has collected information on Norwegian smoking habits as a supplement to the relevant quarterly survey each year, with the aim of providing data on the level and development of smoking behaviour in the population.

Although Statistics Norway conducted all these surveys, they were developed and financed by external actors, including the Tobacco Injury Council, the Directorate of Health, the Norwegian Institute for Alcohol and Drug Research, and the Norwegian Institute of Public Health.

Table S1 summarizes the survey questions employed. We derived the smoking status using the variables tob1_hender (**Do you ever smoke**), tob2_daglig (**Do you smoke daily or occasionally**), and royk_tidligere_daglig (**Have you ever smoked daily**). We classified as daily never smokers the individuals who reported not current smoking (tob1_hender == 2) and no history of daily smoking (royk_tidligere_daglig == 0). We classified as daily current smokers those who reported currently smoking (tob1_hender == 1 and tob2_daglig == 1), and we classified daily former smokers as those who did not currently smoke but had previously smoked daily (tob1_hender == 2 & royk_tidligere_daglig == 1), and we excluded from this analysis those who reported to smoke occasionally (tob1_hender == 1 and tob2_daglig == 2). Only daily smokers were included in the analysis. This categorization allows for consistent identification of smoking history across the population. We did not include questions on other tobacco products in the analysis, such as snus, and we didn’t distinguish between pipes, cigars, cigarillos.

Figure S1. Trends in daily smoking prevalence in Norway and major tobacco control policies, 1973–2024. The figure shows the decline in daily smoking prevalence among men and women in Norway from 1973 to 2024, alongside the timing of major tobacco control measures. Smoking prevalence decreased substantially over the study period in both sexes, with convergence between men and women occurring from the late 1990s onward.

Figure S2. Age, period, and cohort ranges used in the analysis. The earliest birth cohort included is 1904, and the most recent cohort providing cessation data is 2008. We assumed cessation probabilities to be zero before age 15 (black dashed line), and initiation probabilities to be zero before age 8 (red dashed line). NIPH data were available for ages 16–79 and years 1973–2024 (yellow box). Model-based projected estimates are shown in blue.

| **Table S1. Survey data and questionnaires on tobacco use.** | |
| --- | --- |
| **Year stopped smoking daily** | Når sluttet du å røyke daglig?  When did you stop smoking daily? |
| **Age started smoking daily** | Hvor gammel var du da du begynte å røyke daglig?  How old were you when you started smoking daily? |
| **Number of cigarettes smoked daily** | Hvor mange sigaretter røyker du gjennomsnittlig pr.dag?  Fabrikklagede og hjemmerullede.  How many cigarettes do you smoke per day on average?  Factory-made and home rolled. |
| **Sometimes smokes** | Hender det at du røyker?  Do you ever smoke? |
| **Smokes daily or occasionally** | Røyker du daglig eller av og til?  Do you smoke daily or occasionally? |
| **Used to smoke daily** | Har du noen gang røykt daglig?  Have you ever smoked daily? |

| **Table S3. Distributions of sex and age by survey year** | | | | |
| --- | --- | --- | --- | --- |
|  | Men | | Age | |
| Year | Mean | Standard deviation | Mean | Standard deviation |
| 1973 | 0.501 | 0.500 | 44.584 | 16.697 |
| 1976 | 0.492 | 0.500 | 43.895 | 16.524 |
| 1977 | 0.493 | 0.500 | 43.646 | 16.715 |
| 1978 | 0.493 | 0.500 | 43.424 | 16.550 |
| 1979 | 0.493 | 0.500 | 43.076 | 16.414 |
| 1980 | 0.493 | 0.500 | 42.679 | 16.460 |
| 1981 | 0.498 | 0.500 | 42.703 | 16.597 |
| 1982 | 0.490 | 0.500 | 42.421 | 16.579 |
| 1983 | 0.504 | 0.500 | 43.002 | 16.660 |
| 1984 | 0.498 | 0.500 | 42.283 | 16.242 |
| 1985 | 0.495 | 0.500 | 42.264 | 16.530 |
| 1986 | 0.513 | 0.500 | 42.223 | 16.464 |
| 1987 | 0.501 | 0.500 | 42.123 | 16.451 |
| 1988 | 0.504 | 0.500 | 42.179 | 16.308 |
| 1989 | 0.498 | 0.500 | 42.336 | 16.184 |
| 1990 | 0.496 | 0.500 | 42.521 | 16.476 |
| 1991 | 0.491 | 0.500 | 41.560 | 16.042 |
| 1992 | 0.528 | 0.499 | 42.784 | 16.101 |
| 1993 | 0.511 | 0.500 | 41.966 | 15.951 |
| 1994 | 0.483 | 0.500 | 41.844 | 15.729 |
| 1995 | 0.496 | 0.500 | 42.333 | 15.960 |
| 1996 | 0.505 | 0.500 | 41.612 | 15.644 |
| 1997 | 0.493 | 0.500 | 41.824 | 15.469 |
| 1998 | 0.500 | 0.500 | 41.926 | 15.329 |
| 1999 | 0.502 | 0.500 | 41.971 | 15.293 |
| 2000 | 0.507 | 0.500 | 42.214 | 15.505 |
| 2001 | 0.488 | 0.500 | 42.847 | 15.578 |
| 2002 | 0.507 | 0.500 | 42.947 | 15.482 |
| 2003 | 0.499 | 0.500 | 42.554 | 15.680 |
| 2004 | 0.497 | 0.500 | 42.292 | 15.649 |
| 2005 | 0.500 | 0.500 | 42.796 | 15.680 |
| 2006 | 0.504 | 0.500 | 42.942 | 15.757 |
| 2007 | 0.497 | 0.500 | 44.209 | 15.385 |
| 2008 | 0.493 | 0.500 | 44.345 | 15.474 |
| 2009 | 0.492 | 0.500 | 44.747 | 15.516 |
| 2010 | 0.493 | 0.500 | 44.963 | 15.518 |
| 2011 | 0.494 | 0.500 | 45.013 | 15.922 |
| 2012 | 0.493 | 0.500 | 45.657 | 16.011 |
| 2013 | 0.511 | 0.500 | 45.538 | 16.054 |
| 2014 | 0.510 | 0.500 | 44.620 | 16.096 |
| 2015 | 0.513 | 0.500 | 44.217 | 15.953 |
| 2016 | 0.507 | 0.500 | 44.338 | 16.122 |
| 2017 | 0.510 | 0.500 | 44.303 | 16.259 |
| 2018 | 0.513 | 0.500 | 44.408 | 16.117 |
| 2019 | 0.509 | 0.500 | 44.434 | 16.385 |
| 2020 | 0.509 | 0.500 | 44.934 | 16.374 |
| 2021 | 0.509 | 0.500 | 44.650 | 16.289 |
| 2022 | 0.508 | 0.500 | 44.909 | 16.425 |
| 2023 | 0.510 | 0.500 | 44.749 | 16.403 |
| 2024 | 0.504 | 0.500 | 44.875 | 16.485 |

| **Table S4. Number of respondents by survey year** | | | | | |
| --- | --- | --- | --- | --- | --- |
|  | Quarter | | | |  |
| Year | 1 | 2 | 3 | 4 | Total |
| 1973 | 0 | 0 | 0 | 2313 | 2313 |
| 1974 | 0 | 0 | 0 | 9667 | 9667 |
| 1975 | 0 | 0 | 0 | 7930 | 7930 |
| 1976 | 0 | 0 | 0 | 2341 | 2341 |
| 1977 | 0 | 0 | 0 | 2372 | 2372 |
| 1978 | 0 | 0 | 0 | 2475 | 2475 |
| 1979 | 0 | 0 | 0 | 2560 | 2560 |
| 1980 | 0 | 0 | 0 | 2421 | 2421 |
| 1981 | 0 | 0 | 0 | 2548 | 2548 |
| 1982 | 0 | 0 | 0 | 2597 | 2597 |
| 1983 | 0 | 0 | 0 | 2386 | 2386 |
| 1984 | 0 | 0 | 0 | 2564 | 2564 |
| 1985 | 0 | 0 | 0 | 2449 | 2449 |
| 1986 | 0 | 0 | 0 | 2574 | 2574 |
| 1987 | 0 | 0 | 0 | 2792 | 2792 |
| 1988 | 0 | 0 | 0 | 2446 | 2446 |
| 1989 | 0 | 0 | 0 | 2467 | 2467 |
| 1990 | 0 | 0 | 0 | 2537 | 2537 |
| 1991 | 0 | 0 | 0 | 2588 | 2588 |
| 1992 | 0 | 0 | 0 | 1890 | 1890 |
| 1993 | 0 | 0 | 0 | 1778 | 1778 |
| 1994 | 0 | 1505 | 0 | 1951 | 3456 |
| 1995 | 1474 | 1355 | 1052 | 1411 | 5292 |
| 1996 | 1316 | 1393 | 1334 | 1346 | 5389 |
| 1997 | 1443 | 1377 | 1300 | 1313 | 5433 |
| 1998 | 1342 | 1226 | 1400 | 1329 | 5297 |
| 1999 | 1261 | 1416 | 1287 | 1162 | 5126 |
| 2000 | 1471 | 1395 | 1236 | 1122 | 5224 |
| 2001 | 1375 | 1336 | 1295 | 1274 | 5280 |
| 2002 | 1465 | 1201 | 1413 | 1439 | 5518 |
| 2003 | 1331 | 1275 | 1386 | 1309 | 5301 |
| 2004 | 1362 | 1235 | 1323 | 1347 | 5267 |
| 2005 | 1440 | 1235 | 1297 | 1278 | 5250 |
| 2006 | 1292 | 1195 | 1291 | 1217 | 4995 |
| 2007 | 1287 | 1212 | 1279 | 1248 | 5026 |
| 2008 | 1289 | 1120 | 1122 | 1136 | 4667 |
| 2009 | 1274 | 1161 | 1110 | 1206 | 4751 |
| 2010 | 1240 | 1014 | 1069 | 1072 | 4395 |
| 2011 | 1215 | 1135 | 1143 | 1158 | 4651 |
| 2012 | 1226 | 1017 | 1193 | 1198 | 4634 |
| 2013 | 1253 | 1091 | 1155 | 1123 | 4622 |
| 2014 | 1257 | 1075 | 1082 | 1175 | 4589 |
| 2015 | 1178 | 2242 | 1202 | 1941 | 6563 |
| 2016 | 1207 | 2068 | 1161 | 1620 | 6056 |
| 2017 | 1225 | 2197 | 1104 | 1742 | 6268 |
| 2018 | 1138 | 2185 | 1065 | 1723 | 6111 |
| 2019 | 1143 | 2145 | 1079 | 1678 | 6045 |
| 2020 | 1137 | 2189 | 1174 | 1697 | 6197 |
| 2021 | 1186 | 2185 | 965 | 1534 | 5870 |
| 2022 | 1068 | 1947 | 1020 | 1412 | 5447 |
| 2023 | 988 | 2123 | 1109 | 1109 | 5329 |
| 2024 | 0 | 4954 | 0 | 0 | 4954 |
| Total |  |  |  |  | 226698 |

# II. Methodology Overview for the Age-Period-Cohort Model

**Summary**

We used Age-Period-Cohort (APC) models to estimate temporal patterns of cigarette smoking for the Norwegian population. These models explicitly separate the contributions of age, period (calendar year), and birth cohort to trends in smoking prevalence and related behaviors. Formally, the expected prevalence of smoking at age $i$ and calendar year *j* is defined as:

*logit(Previ,j)=log(A_i_)+log(P_j_)+log(C_k_),*

where *A_i_* represents age effects, *P_j_* period effects, and *C_k_* cohort effects (*k = j−i*). The logit denotes the natural log of the odds. Continuous age (as single years), period, and cohort effects were modeled using constrained natural splines. [3, 4]

The modeling framework assumes three compartments: never smokers, current smokers, and former smokers (daily smoking). Individuals may initiate smoking (transitioning from never to current smoker) or cease (transitioning from current to former smoker). Initiation probabilities were estimated from reported age at smoking initiation, expressed as age-specific conditional probabilities among never smokers. To reduce recall bias and account for differential mortality between smokers and never smokers, cumulative initiation probabilities were matched by cohort to the estimated ever-smoking prevalence at age 30.

Cessation probabilities were estimated as age-specific conditional probabilities of cessation among daily current smokers at interview. For each sex and cohort, age-specific current smoking prevalence was derived by multiplying ever-smoking prevalence by the proportion of smokers who had not ceased at that age. Former smoking prevalence was obtained as the difference between ever smoking and current smoking prevalence at each age.

**Introduction**

This supplementary material provides the mathematical details underlying the estimation of initiation and cessation probabilities, smoking prevalence, and smoking intensity (cigarettes per day) by sex using Age–Period–Cohort (APC) models. Our approach follows Holford et al.[4], with modifications to allow sex-specific estimation. Separate APC models with consistent specifications (e.g., spline knots and parameterization) were fitted for females and males to obtain sex-specific smoking parameters. Details of each fitted model are provided below. The final placement of knots was guided by face validity and expert judgment. Both the knot locations and the model structure, including the choice between age–period–cohort and age–cohort specifications, were varied and examined in sensitivity analyses before the final models were selected.

A schematic of the compartment model used to represent smoking histories is shown in Figure S1. In this framework, individuals may initiate smoking at some age and may subsequently cesed. Although this representation simplifies the complexity of actual smoking behaviors, it provides a useful approximation for most of the population. Modeling cessation is particularly challenging, as cessation attempts are often unsuccessful. To address this, we adopted the convention that individuals reporting cessation must have remained abstinent for at least two years prior to interview. Otherwise, the observation period was truncated at the reported age of cessation, and the individual was reclassified as a current smoker at that age. Sensitivity analyses varying the abstinence threshold (0–5 years) showed consistent results for thresholds up to 3 years, whereas longer thresholds (4–5 years) produced implausible increases in smoking prevalence after age 30 and were therefore excluded.

Figure S3. Model compartments for smoking history. Dashed lines represent the ever-smoking population.

Never smoker

-

Current smoker

Former smoker

Dead

We defined the basic quantities of interest conditional on a hypothetical scenario without transitions to death. Let *a* denote age, *t* period (calendar year), and *c* cohort (year of birth). All three temporal dimensions influence the construction of the parameters governing smoking history. Since these indicators are related by $c=t-a$ , the relationships among the model parameters can, without loss of generality, be expressed as functions of age and cohort. The smoking initiation probability, $p\left( a,c \right)$, is defined as the conditional probability of initiating smoking at age $a$ for cohort $c$, given that the individual was not a smoker at age *a-1*, i.e.,

$p\left( a,c \right)=\Pr\left\{ \text{Smoker at }a\left| \text{Not smoker at }\left( a-1 \right),c \right. \right\}$

It is related to the cumulative proportion of ever smokers at *a* conditional on remaining alive,

$P_{E}(a,c)=1-\prod_{i=1}^{a} \left[ 1-p\left( i,c \right) \right]=1-[{1-P}_{E}\left( a-1,c \right)][1-p\left( a,c \right)]$

where $P_{E}\left( 0,c \right)=0$, which is equivalent to the actuarial approach for estimating the survival curve.

If smoking had no effect on mortality, then equation , which is conditional on survival, would also hold in a population observed over time. Smoking influences mortality, such that the observed proportion of ever smokers at age $a$in cohort $c$, denoted$P_{E}^{*}\left( a,c \right)$, satisfies $P_{E}^{*}\left( a,c \right)\leq P_{E}\left( a,c \right)$. Initiation probabilities estimated from survey data are similarly affected by differential mortality. We represent these as $p^{*}\left( a,c \right)=p(a,c)/C_{p}$ where $C_{p}\geq1$ is a correction factor introduced to adjust for this bias.

We assumed that differential mortality among smoking categories is negligible at younger ages but becomes increasingly influential with age. For example, in early cohorts where survey data are available only for ages above 30, differential mortality is expected to introduce substantial downward bias in unadjusted initiation estimates, $\hat{p}^{*}\left( a,c \right)$. In more recent cohorts, nearly all initiation occurs before age 30; however, in cohorts born earlier in the twentieth century, later initiation—particularly among females—was not uncommon, thereby delaying the onset of differential mortality effects. Accordingly, we assumed that smoking-related differential mortality begins to act at ages $a\geq a_{0}$, such that $P_{E}^{*}\left( a,c \right)=P_{E}\left( a,c \right)$ for $a<a_{0}$. Corrected initiation probabilities were then obtained by solving

$P_{E}\left( a_{0},c \right)=1-\prod_{i=1}^{a_{0}} \left[ 1-C_{p}p^{*}\left( i,c \right) \right]$

for $C_{p}$, i.e., by matching the cumulative initiation rates to the estimated prevalence at age $a_{0}$. We assumed that $a_{0}$ was the age 30, because the mortality difference between smokers and non-smokers has not yet taken effect. The estimated transition probabilities should be interpreted as approximations to the underlying mortality-free behavioral process, with residual bias potentially remaining at older ages due to incomplete adjustment for differential mortality.

Smoking cessation was assumed to be a function of age for each cohort. The smoking cessation probability conditional on the subject being alive and currently smoking is

$q\left( a,c \right)=\Pr\left\{ \text{Former smoker at }a\left| \text{Smoker at }\left( a-1 \right),c \right. \right\}$.

We assumed that $q\left( a,c \right)=0$ for $a<15$ and we estimated it for $15\leq a\leq99$. The cumulative proportion of smokers in cohort c who had not ceased smoking by age *a* is given by

$Q\left( a,c \right)=\prod_{i=15}^{a} \left[ 1-q\left( i,c \right) \right]$

For simplicity, we assumed that cessation probabilities do not depend on age at smoking initiation, cigarettes per day, or other individual-level factors potentially influencing cessation success. Because initiation typically occurs within a relatively narrow age range, variation in age of initiation becomes less consequential for mortality as cohorts age. Incorporating smoking intensity into a cessation model would require detailed lifetime smoking histories, which were not systematically collected in the Travel and Holiday Surveys (Omnibus) or the Smoking Habits Surveys.

Current smokers represent ever smokers who have not ceased, and given our assumption that this only depends on age for a given cohort, the prevalence is

$P_{C}\left( a,c \right)=P_{E}\left( a,c \right)Q\left( a,c \right)$

Former smokers are those who have smoked at some point in their lives, but cease before age *a*, and the proportion of these individuals is

$P_{F}\left( a,c \right)=P_{E}\left( a,c \right)-P_{C}\left( a,c \right)=P_{E}(a,c)[1-Q\left( a,c \right)]$

Finally, the proportion of cohort *c* who have never smoked is the complement of those who ever smoked,

$P_{N}\left( a,c \right)=1-P_{E}\left( a,c \right)$

For a given age and cohort, the sets of current, former and never smokers are exhaustive, i.e.,

$P_{C}\left( a,c \right)+P_{F}\left( a,c \right)+P_{N}\left( a,c \right)=1$

**Estimation of smoking parameters**

Data were available only for restricted ranges of age, $a\in\left[ \overset{̣}{a},\dot{a} \right]$, and period, $t\in\left[ \overset{̣}{t},\dot{t} \right]$ such that the earliest cohort was $\overset{̣}{c}=\overset{̣}{t}-\dot{a}$ and the latest cohort was $\dot{c}=\dot{t}-\overset{̣}{a}$. For a given cohort c, the observed age range was therefore $a\in\left[ \overset{̣}{t}-c,\dot{t}-c \right]$. To reconstruct smoking histories outside the ranges represented in the surveys, each temporal effect was modeled as a nonparametric function and extrapolated beyond the observed data. For application within a broader decision-support framework to guide future strategies for controlling smoking-related diseases, the model was applied to birth cohorts spanning 1904–2008. Because initiation generally occurs early in life, initiation patterns were better represented in more recent cohorts, whereas cessation occurs across the life course and was better captured in older ages of earlier cohorts. The Travel and Holiday Surveys (Omnibus) and Smoking Habits Surveys contributed data from different life stages, necessitating extrapolation beyond observed ranges to estimate cohort smoking histories across the full lifespan. We incorporated sample adult weights to ensure the estimates were representative of the overall population.

**Cross-sectional estimates of daily ever smokers by sex**

For survey years with information on smoking status and sex (i.e., all years included in the analysis), participants provided data that allowed estimation of the prevalence of daily ever smokers by age *a* for the corresponding cohort, defined as *c=t-a*. Let Y_i_ *=* 1 if the *i*-th individual ever smoked and Y_i_ *=* 0 otherwise, with probability of response given by $P_{E}\left( a,c \right)$, a function of age and cohort. We assumed an additive logistic model for Y_i_, such that:

$\text{logit}\left\{ P_{E}\left( a,c \right) \right\}=\beta_{0}+\beta_{a}\left( a \right)+\beta_{c}\left( c \right)$

where $\beta_{0}$ is an intercept and $\beta_{\cdot}\left( \cdot\right)$ is a function given by a constrained natural spline.[5]

For males, we fitted an age-cohort model using “survey” package in R (version 4.5.1) with knots specified as

Age: 35,50,60,75

Cohort: 1915,1935,1955

For females, we fitted an age-cohort model with knots specified as

Age: 40,50,70

Cohort: 1950,1970,1990

Age effects were estimated between 30 and 99.

We evaluated model calibration across age×cohort cells by comparing observed and predicted prevalence. The calibration plot showed that most cell-specific estimates lay close to the 45-degree line, with no strong systematic pattern of overprediction or underprediction. Quantitatively, the mean absolute calibration error was 0.050, the root mean squared calibration error was 0.068, and the mean signed calibration error was 0.005, indicating little overall directional bias. Residual overdispersion was present (Pearson scale 1.52), and therefore inference was based on a survey-weighted quasi-binomial model.

**Smoking initiation probability**

Unadjusted age-specific initiation probabilities for a given cohort, denoted $\hat{p}^{*}\left( a,c \right)$, were derived directly from reported age at initiation and sex, which were available in all survey years. For each cohort represented in a survey, we identified the number of individuals who initiated smoking at age $a,d\left( a,c \right)$, and the number who had never smoked up to that age, $n\left( a,c \right)$. These values formed the response data in a logistic regression model, with temporal factors represented by nonparametric functions to be estimated.

Because each survey included only participants who had survived to the time of data collection, cohorts were disproportionately represented by individuals who initiated smoking later in life or never smoked, leading to downward bias in the estimated initiation probabilities. To correct for this, we introduced a correction factor by specifying that the estimated cumulative initiation probability at a given age, $a_{0}$, must equal the value obtained from the cross-sectional analysis, i.e.,

$\hat{P}_{E}\left( a^{*},c \right)=1-\prod_{i=1}^{a^{*}} \left[ 1-\hat{C}_{p}{\times\hat{p}}^{*}\left( i,c \right) \right]$

and finding $\hat{C}_{p}$ which satisfies this condition.

To determine the crude initiation probability estimates, an age-period-cohort model was fitted to the tabulated data given number of subjects who start smoking and are at risk of starting at a given age,

$\text{logit}\left\{ p^{*}\left( a,c \right) \right\}=\beta_{0}+\beta_{a}\left( a \right)+\beta_{t}\left( t \right)+\beta_{c}(c)$

where $\beta_{0}$ is an intercept and $\beta_{\cdot}\left( \cdot\right)$ is given by a constrained natural spline. Knots were specified as:

age: 10,20,30 for females

age: 10,12,14,15,16,17,21,24,27,35 for males

period: 1930,1940,1950,1960,1970,1980,1990,2000,2005,2010,2015

cohort: 1940,1950,1960,1970,1980,1990

Age for the target used to determine the correction factor was age 30. The target value for the cumulative probability of being a smoker was the estimate derived in the analysis of the prevalence curve, $\hat{\Pi}\left( a^{*},c \right)$.

**Smoking cessation probability**

Data for this analysis were obtained from the cleaned datasets of the Travel and Holiday Surveys (Omnibus) and the Smoking Habits Surveys, which included respondents aged 15–80 years. Reported cessation ages younger than 8 were recoded as 8. For each year of age following smoking initiation, a binary outcome variable was defined according to our criterion for cessation. Yearly estimates were then obtained using a logistic age–period–cohort model, with cohort effects constrained to have an intercept of 0 and slope of 0, in which

$\text{logit}\left\{ q\left( a,c \right) \right\}=\beta_{0}+\beta_{a}\left( a \right)+\beta_{t}\left( t \right)+\beta_{c}\left( c \right)$

where $\beta_{0}$ is an intercept and $\beta_{\cdot}\left( \cdot\right)$ are given by a constrained natural spline. We were only interested in the fitted values for the cessation probabilities, which are not affected by the well-known identifiability problem in age-period-cohort models[3]. Knots were specified as follows:

age: 30, 40, 50, 60

period: 1940, 1960, 1980, 2000

cohort: 1930, 1940, 1950, 1960, 1970, 1980, 1990

Estimates of the yearly cessation probability for age $a$ and cohort $c$ were the fitted values for ages 15-99, $\hat{q}\left( a,c \right)$.

**Cigarettes smoked per day (CPD)**

Smoking dose was analyzed as an ordered categorical outcome, with the midpoint of each category taken as the representative value. The half-pack category was typically the modal value and close to the mean. The intervals (approximate midpoints) were: CPD ≤ 5 (3); 5 < CPD ≤ 15 (10); 15 < CPD ≤ 25 (20); 25 < CPD ≤ 35 (30); 35 < CPD ≤ 45 (40); and CPD > 45 (66). Information on cigarettes per day was available in all survey years of the Travel and Holiday Surveys (Omnibus) and the Smoking Habits Surveys.

A cumulative logistic model was fitted using the “*svyVGAM”* package in R. Age, period, and cohort were modeled as additive nonparametric functions of time using constrained natural splines, with knots specified as:

Age knots: 30,40,50,60

Period knots: 2010,2013,2016

Cohort knots: 1950,1970,1990,2000

# III. Comparison of current smoking prevalence and mean CPD as reported by surveys vs model predictions and CPD analysis

To evaluate the accuracy of our estimates, we compared survey-reported current smoking prevalence and mean CPD with corresponding model predictions, stratified by age group and survey period. Figures S2 and S3 present the results for current smoking, and Figures S4 and S5 for mean CPD, among females and males, respectively. Overall, the model reproduced observed patterns of smoking prevalence and mean CPD with satisfactory accuracy, with no substantial deviations evident.

Figure S4. Age-specific female current smoking prevalence by survey period: empirical estimates (circles) versus model predictions (solid line)

Figure S5. Age-specific male current smoking prevalence by survey period: empirical estimates (circles) versus model predictions (solid line)

Figure S6. Age-specific mean CPD for selected birth cohorts between 1950 and 2000, by sex. Each colored line represents a specific birth cohort, starting with the 1950 cohort (light blue line). The progression of the line along the horizontal axis illustrates how mean CPD changes as the corresponding birth cohort ages.

Figure S7. Age-specific mean cigarettes per day (CPD) among females by survey period: survey estimates (circles) versus model predictions (solid line)

Figure S8. Age-specific mean cigarettes per day (CPD) among males by survey period: survey estimates (circles) versus model predictions (solid line)

# IV. Uncertainty Analysis

Figure S9. Initiation probabilities (solid lines) and 90% bootstrap confidence intervals (dotted lines) by gender, calendar year and birth cohort.

Figure S10. Daily ever smoker prevalence (solid lines) and 90% bootstrap confidence intervals (dotted lines) by gender, calendar year and birth cohort.

Figure S11. Cessation probabilities (solid lines) and 90% bootstrap confidence intervals (dotted lines) by gender, calendar year and birth cohort.

Figure S12. Daily current smoker prevalence (solid lines) and 90% bootstrap confidence intervals (dotted lines) by gender, calendar year and birth cohort.

# V. Association between daily smoking and daily snus use

Figure S13. Association between daily smoking and daily snus use in males 16-24 years (“Røyker daglig”= Daily smoking, “Bruker snus daglig”= Daily snus use). Figure from the Norwegian Institute of Public Health[6].

Figure S14. Association between daily smoking and daily snus use in females 16-24 years (“Røyker daglig”= Daily smoking, “Bruker snus daglig”= Daily snus use). Figure from the Norwegian Institute of Public Health[6].

# VI. Overall snus use and current smoking

Snus use in Norway increased substantially from around the year 2000, particularly among cohorts born around 1980, who increasingly chose snus over cigarettes upon initiating tobacco use. However, whether this reflects substitution is uncertain, as some individuals may not have initiated smoking in the absence of snus, consistent with patterns in countries without snus[7]. The low prevalence of daily smoking among young adults in Norway suggests that snus may have contributed to reduced smoking initiation in cohorts born after 1980.

Among Norwegian 16–24-year-olds, daily smoking declined from 13% in 2010 to 5% in 2020, compared with a more modest decrease in Denmark (15–29 years), from approximately 11% to 10%[8]. Snus has also been associated with higher cessation probabilities, however, causality remains unclear, as users may have been more likely to cease regardless [9].

More generally, substitution between nicotine products is well documented, particularly following the emergence of alternative products such as e-cigarettes, suggesting that shifts from cigarettes to other nicotine sources are not unique to snus or the Norwegian context[10]. Cross-country comparisons indicate that while snus may have contributed to declining smoking prevalence, its overall impact appears limited. In Denmark, daily smoking fell from approximately 38% in 1994 to 16% in 2017, compared with a decline from 34% to 11% in Norway, suggesting broadly similar trends.

Figures S13 and S14 present trends in daily smoking and snus use, while Figure S1 shows temporal changes in overall smoking prevalence alongside the introduction of major tobacco control measures. These figures are included to provide historical and population-level context for changes in tobacco use patterns over time and are not intended to imply causal relationships between specific policies/snus consumption and observed trends.

# VI. References

1.	*Travel and Holiday Survey.*

2.	*Smoking Habits Survey.*

3.	Holford, T.R., D.T. Levy, and R. Meza, *Comparison of Smoking History Patterns Among African American and White Cohorts in the United States Born 1890 to 1990.* Nicotine Tob Res, 2016. **1**(Suppl 1).

4.	Holford, T.R., D.T. Levy, and L.A. McKay, *Patterns of birth cohort-specific smoking histories, 1965-2009.* Am J Prev Med. Feb, 2014. **46**(2).

5.	Durrleman, S. and R. Simon, *Flexible regression models with cubic splines.* Stat Med, 1989. **8**(5): p. 551–561.

6.	*Utbredelse av røyking i Norge*.

7.	Health, W.K.E.K.N.L.S.C.W.A.t.W.C.A.J.P., *Nov;114(11):1191-1194.* 2024.

8.	*Danskernes rygevaner, Udviklingen fra 1994 til 2017, December 2018, Elektronisk udgave.*

9.	Lund, I. and S.G. Christiansen, *Association between snus use over time and smoking cessation in Norwegian smokers.* Addiction, 2020. **115**: p. 170–174.

10.	Huang, J., Gwarnicki, C., Xu, X., Caraballo, R. S., Wada, R., & Chaloupka, F. J., *A comprehensive examination of own- and cross-price elasticities of tobacco and nicotine replacement products in the U.S. Preventive Medicine.* 2018.
